# Metabolic profiling of ligands for the chemokine receptor CXCR3 by liquid chromatography-mass spectrometry coupled to bioaffinity assessment

**DOI:** 10.1007/s00216-015-8867-z

**Published:** 2015-07-12

**Authors:** Marija Mladic, Danny J. Scholten, Maikel Wijtmans, David Falck, Rob Leurs, Wilfried M. A. Niessen, Martine J. Smit, Jeroen Kool

**Affiliations:** Division of BioAnalytical Chemistry, Amsterdam Institute for Molecules Medicines and Systems, VU University Amsterdam, De Boelelaan 1083, 1081HV Amsterdam, The Netherlands; Division of Medicinal Chemistry, Amsterdam Institute for Molecules Medicines and Systems, VU University Amsterdam, De Boelelaan 1083, 1081HV Amsterdam, The Netherlands; hyphen MassSpec, de Wetstraat 8, 2332 XT Leiden, The Netherlands

**Keywords:** Liquid chromatography- mass spectrometry (LC-MS), At-line nanofractionation, Bioaffinity assessment, Metabolic profiling, Drug metabolism, CXCR3

## Abstract

Chemokine receptors belong to the class of G protein-coupled receptors and are important in the host defense against infections and inflammation. However, aberrant chemokine signaling is linked to different disorders such as cancer, central nervous system and immune disorders, and viral infections [Scholten DJ et al. (2012) Br J Pharmacol 165(6):1617–1643]. Modulating the chemokine receptor function provides new ways of targeting specific diseases. Therefore, discovery and development of drugs targeting chemokine receptors have received considerable attention from the pharmaceutical industry in the past decade. Along with that, the determination of bioactivities of individual metabolites derived from lead compounds towards chemokine receptors is crucial for drug selectivity, pharmacodynamics, and potential toxicity issues. Therefore, advanced analytical methodologies are in high demand. This study is aimed at the optimization of a new analytical method for metabolic profiling with parallel bioaffinity assessment of CXCR3 ligands of the azaquinazolinone and piperazinyl-piperidine class and their metabolites. The method is based on mass spectrometric (MS) identification after liquid chromatographic (LC) separation of metabolic mixtures. The bioaffinity assessment is performed “at-line” via high-resolution nanofractionation onto 96-well plates allowing direct integration of radioligand binding assays. This new method enables identification of metabolites from lead compounds with associated estimation of their individual bioaffinity. Moreover, the identification of the metabolite structures via accurate mass measurements and MS^2^ allows the identification of liable metabolic “hotspots” for further lead optimization. The efficient combination of chemokine receptor ligand binding assays with analytical techniques, involving nanofractionation as linking technology, allows implementation of comprehensive metabolic profiling in an early phase of the drug discovery process.

**Electronic supplementary material:**

The online version of this article (doi:10.1007/s00216-015-8867-z) contains supplementary material, which is available to authorized users.

## Introduction

In general, the majority of drugs undergo in vivo metabolism to more polar products leading to improved clearance by the kidney. During this biotransformation process, even subtle changes in the structure may lead to changes in the bioactivity at and/or affinity for the drug target. Moreover, the selectivity and functional activity of these metabolites can be seriously compromised compared to the parent compound. This may influence the therapeutic effect of the drug and, as a consequence, prevent a lead compound from being developed further. In that respect, determination of the bioactivity of individual metabolites derived from lead candidates is crucial for profiling the eventual pharmacodynamics of the drug and highlighting potential toxicity issues. These studies are usually restricted to only a few lead candidates and postponed until a later stage of drug development since currently used analytical methods are laborious and costly and do not allow bioactivity assessment of metabolites, but only their identification. However, metabolic profiling of a lead candidate in an early stage of the drug discovery process is highly desirable as attrition costs increase when drug candidates progress through the drug discovery and development stages [2].

Recently, a new analytical strategy that allows characterization of bioactive metabolites from G protein-coupled receptor (GPCR) ligands at an early stage of lead optimization [3] was introduced. In this regard, bioactivity is referring to the interaction of such a metabolite with the receptor displayed as, e.g., receptor binding affinity and/or receptor (in)activation. In a parallel study, the histamine H_4_ receptor was considered the drug target while metabolite binding to the histamine H_3_ receptor was incorporated to assess selectivity loss by off-target binding to the H_3_ receptor [4]. The analytical technology is based on mass spectrometric (MS) identification of individual metabolites after liquid chromatographic (LC) separation of metabolic mixtures. The parallel assessment of bioactivity is performed at-line via high-resolution nanofractionation onto high-density microtiter plates followed by performing either receptor binding or functional assays [3, 4]. Since the high-resolution nanofractionation collects fractions in the second range, the eventual bioactivities measured from the wells can be reconstructed into bioactivity chromatograms with sufficient resolution to allow accurate correlation of bioactivity to individual metabolites. To this end, the peak shapes and retention times from the reconstructed bioactivity chromatograms are compared with the LC-MS data from which the bioactives are identified.

This study is aimed at the optimization of a new analytical method for bioaffinity assessment of mixtures of small molecule ligands binding the CXCR3 receptor, such as metabolic mixtures. CXCR3 is a member of the chemokine receptor family that binds chemokines, which are small proteins ranging between 8 and 12 kDa, as their endogenous ligands [5]. Chemokine receptors in turn belong to the superfamily of G protein-coupled receptors (GPCRs). GPCRs are 7-transmembrane spanning receptor proteins that play a key role in different physiological and pathological conditions and have demonstrated to be valid drug targets. The chemokine receptor CXCR3, for example, is a potential drug target for several immune-related diseases, such as allograft rejection [6], atherosclerosis [7], psoriasis [8], multiple sclerosis [9, 10], rheumatoid arthritis [11], and type I diabetes [12] where aberrant CXCR3 and ligand expression seems to be a common denominator [13, 14]. Moreover, antagonism of CXCR3 in animal models of disease showed beneficial effects in reduction of allograft rejection in organ transplantation as well as attenuation of atherosclerotic plaques and metastasis of certain tumors [15–19]. As such, development of small molecule drugs targeting the CXCR3 receptor attracted the interest of the pharmaceutical industry and academia. Their efforts have led to the discovery and development of high-affinity small molecule ligands for the CXCR3 receptor by high-throughput screening of libraries of compounds and natural products [13, 14]. The different classes of CXCR3 ligands have been reviewed [14]. One well-studied class of compounds is the (aza)quinazolinone class [13, 14], which includes AMG487 [20], one of the early CXCR3 antagonists that showed therapeutic potential. It even entered clinical trials for treatment of psoriasis and progressed to phase II, yet it was discontinued due to lack of efficacy. It has been suggested that this clinical failure is caused by non-linear pharmacokinetics due to inhibition of cytochrome P450 enzymes (CYP3A) resulting in variable drug exposure [21, 22]. Another well-studied compound from this class is NBI-74330 [23], which has higher affinity than AMG487 and also shows therapeutic potential in an animal model of atherosclerosis [18].

The piperazinyl-piperidine class of compounds represents another distinct chemical class containing high-affinity allosteric CXCR3 antagonists [24–28]. Interestingly, a compound from this class (SCH-546738) shows activity in several animal models of disease, including allograft rejection and rheumatoid arthritis [19].

**Fig. 1:**
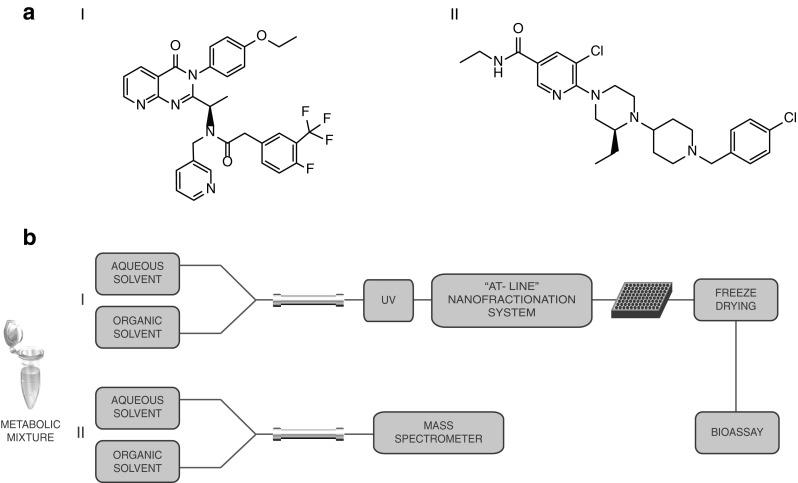
**a** Chemical structures of two CXCR3 antagonists: *I* NBI-74330 from the (aza)quinazolinone class *II* VUF11211 from the piperazinyl-piperidine class. **b** Schematic overview of the analytical setup. *I* Separation and UV detection of the metabolic mixtures is followed by high-resolution nanofractionation for bioaffinity screening in radioligand binding assay. *II* Separation on LC-UV system is followed by accurate MS and MS^2^ measurements for (partial) structure identification of the metabolites present in the mixture

In this study, the analytical method presented combining LC-MS analysis and parallel at-line nanofractionation with subsequent bioaffinity assessment was optimized and validated for analysis of mixtures targeting the CXCR3 chemokine receptor. Furthermore, the optimized method was applied to the metabolic profiling and the bioaffinity assessment of metabolites of two high-affinity small molecule CXCR3 antagonists: NBI-74330 from the azaquinazolinone class [29, 30] and VUF11211 from the piperazinyl-piperidine class [28] (see Fig. 1a for their structures). Both compound classes bind to CXCR3 in an allosteric fashion, having distinct yet partially overlapping binding sites in its transmembrane region [25, 28]. Metabolites were separated using reversed phase liquid chromatography (LC) followed, in parallel, by high-resolution nanofractionation onto 96-well plates to assess the bioaffinity of parent compounds and metabolites and accurate mass MS and MS^2^ analysis for structural identification of formed metabolites. The bioaffinity was assessed using a radioligand binding assay employing the recently described small molecule radioligand [^3^H]-VUF11211 [31]. In total, ten metabolites of NBI-74330 and eight metabolites of VUF11211 have been identified and their structures were partially or fully elucidated. One metabolite of NBI-74330 and two metabolites of VUF11211 were identified as the bioactive metabolites. The active metabolite of NBI-74330 has been already described previously as a pyridyl *N*-oxide in the study conducted by Jopling et al. [32], and its structure and the bioactivity were confirmed in this study. However, no other information about the metabolites of NBI-74330 have been published previously. The metabolism of the VUF11211 compound has not been studied before and the data reported in this study represents the first data known about VUF11211 metabolites so far. Considering the results obtained, this study shows that the efficient combination of receptor ligand binding assays with analytical techniques involving nanofractionation of metabolites is of importance for the implementation of comprehensive metabolic profiling in an early phase of the drug discovery process.

## Material and methods

### Chemicals, reagents, and materials

All the salts used for buffer preparation were of analytical grade and purchased from standard suppliers (Merck, Fluka or Sigma-Aldrich). Cell culture material was obtained from PAA Laboratories GmbH (Pasching, Austria). Glucose-6-phosphate and glucose-6-phosphate-dehydrogenase were purchased from Sigma-Aldrich (Zwijndrecht, The Netherlands). Acetonitrile (ACN; LC-MS grade) was purchased from Biosolve B.V. (Valkenswaard, The Netherlands). Magnesium chloride hexahydrate was purchased from Fluka (Zwijndrecht, The Netherlands). Formic acid (FA) was purchased from Merck (Zwijndrecht, The Netherlands). Nicotinamide adenine dinucleotide phosphate (NADPH) tetra sodium salt was obtained from Applichem (Lokeren, Belgium). G418 was purchased from Duchefa (Haarlem, The Netherlands). ^125^I- CXCL10, [^3^H]-VUF11211 (38.4 Ci/mmol) [31], and Whatman GF/C filter plates were purchased from Perkin Elmer (Groningen, The Netherlands).

VUF11211 was synthesized in enantiopure form in our labs according to the general synthetic procedures patented by Merck [33]. The synthesis of NBI-74330 has been described before [29]. Stock solutions of the ligands were prepared in DMSO (20 mM) and stored at −20 °C.

HEK293 cells stably expressing human CXCR3 receptor (HEK293/CXCR3) were a gift from Dr. K. Biber, University Medical Center Groningen, The Netherlands.

### Liquid chromatography with high-resolution at-line nanofractionation

Liquid chromatography with high-resolution nanofractionation was done in similar fashion as by a recent publication [4]. Briefly, the gradient LC system consisted of a Gilson International (Den Haag, the Netherlands) model 234 autoinjector with a 100-μL loop, a Shimadzu (‘s Hertogenbosch, the Netherlands) LC-20 AD binary pump, and a Shimadzu SPD-20A UV-Vis detector set to 254 nm. The chromatographic column used was a Waters (Milford, MA, USA) xBridge C18 column (4.6 mm × 100 mm; 5 μm) with an xBridge C18 guard column (2.1 mm × 10 mm; 3.5 μm). The LC column was thermostated by a Jones Chromatography (Lakewood, CO, USA) 7971 Column Heater set at 30 °C. The flow rate was 0.6 mL/min. The mobile phase A contained 98 % H_2_O, 2 % ACN, and 0.1 % FA, and the mobile phase B contained 2 % H_2_O, 98 % ACN, and 0.1 % FA. For bioassay optimization, the following gradient was used: 3 min at 0 % B, a linear gradient to 90 % B in 10 min, and 5 min at 90 %. Re-equilibration to starting conditions occurred in 1 min followed by a 4 min isocratic operation. The gradient used for the analysis of metabolic mixtures of NBI-74330 was: 0–3 min at 0 % B, linear gradient to 20 % B in 20 min, 10 min at 20 % B, re-equilibration to 0 % B in 1 min followed by 6 min isocratic separation. Metabolic mixtures of VUF11211 were analyzed starting with 3 min isocratic separation at 25 % followed by 20 min linear gradient to 45 % B and 10 min isocratic separation at 45 % B. Returning to the starting conditions was done in 1 min and followed by 6 min re-equilibration. Nanofractionation of relevant sections of the chromatograms (range where the metabolites and parent eluted) was done directly after the post-column UV detection.

### Liquid chromatography-mass spectrometry

LC-MS analysis of metabolic mixtures was performed using a Shimadzu LCMS ion-trap–time-of-flight hybrid (IT-TOF) system equipped with an electrospray ionization (ESI) source. The injection volume was 50 μL. The separation was performed using the same columns, solvent, and gradient conditions as described above. All MS measurements were performed in positive-ion mode. The instrument settings were: 4.5 kV spray voltage, 125 °C source heating block and curved desolvation line temperature, 70 kPa drying gas pressure, and 1.5 L/min nebulizing gas flow. The scan range was set between *m*/*z* 200 and 1200 with a 10-ms ion accumulation time in MS^2^ data-dependent mode. For CID, the collision energy was set to 90–110 % and collision gas to 50 % at 45 kHz. Shimadzu LCMS Solutions software was used for data acquisition and processing. A Shimadzu SPD-20A UV detector (254 nm) was placed in series prior to the MS for additional data collection on the metabolites (data not used) and for possible transfer to subsequent LC-UV analysis.

### Metabolic incubations

A modified protocol described by Reinen et al. [34] was used for microsomal incubations. Pig liver microsomes (∼20 mg/mL protein) were diluted 1:10 in incubation buffer (50 mM KH_2_PO_4_ buffer pH 7.4, 5 mM MgCl_2_, 5 mM glucose-6-phosphate, and 5 activity units/mL glucose-6-phosphate dehydrogenase) at 4 °C. Ligands were added at room temperature to a final concentration of 100 μM (from 20 mM DMSO stocks). Directly before the start of the incubations, a freshly prepared solution of 30 mM NADPH in 50 mM KH_2_PO_4_ buffer was added at 37 °C in an amount to obtain a 10 % [*v*/*v*] share of the total incubation volumes. The same volume of 10 mM NADPH was added to the incubations 30 and 60 min after the start of the incubations. The incubations were stopped after 90 min with 200 % incubation volume of ice-cold acetonitrile. Subsequently, the formed protein precipitates were centrifuged for 5 min at 11.200*g*. Next, the supernatants were transferred to new Eppendorf tubes for solvent evaporation in a Savant (Holbrook, NY, USA) SpeedVac Plus SC110A. The samples were stored at −20 °C prior to use and re-dissolved in eluent A so that the concentration corresponded to 100 μM of the parent compound prior to incubation.

### Cell culture

A HEK293/CXCR3 cell line was cultured in Dulbecco’s Modified Eagles Medium with high glucose concentration (4.5 g/L) supplemented with 10 % fetal bovine serum, 1 % penicillin/streptomycin, and 400 μg/L G418 at 37 °C in 5 % CO_2_.

### Membrane preparation

The HEK293/CXCR3 crude membrane extracts were prepared as previously described by Verzijl et al. [30]. Briefly, HEK293/CXCR3 cells were washed twice with ice-cold PBS and centrifuged 10 min at 1500*g*. The cell pellets were resuspended in ice-cold membrane buffer (15 mM Tris-HCl, 0.3 mM EDTA, and 2 mM MgCl_2_ at pH 7.4 and 4 °C). After homogenization using a 15-mL Teflon glass homogenizer and rotor, two freeze/thaw cycles were performed. The homogenate was then centrifuged at 40.000*g* for 25 min at 4 °C. The supernatant was carefully removed, and the pellet was resuspended in ice-cold Tris-sucrose buffer (20 mM Tris, 250 mM sucrose at pH 7.4 and 4 °C). The membrane preparation was frozen in liquid nitrogen and stored at −80 °C. The protein concentration was determined using the BCA Protein Assay Kit (Thermo Scientific, Rockford, IL, U.S.A) according to the manufacturer’s instructions.

### Radioligand displacement binding assay

For optimization of the radioligand binding assay, a standard mixture of NBI-74330 and VUF11211 (both at 20 μM concentrations) was separated using the LC conditions described above, and collected onto 96-well plates. On every 96-well plate used for fractionation, two or three rows of outer wells were used to construct IC_50_ curves with internal controls for assessment of maximum and minimum signal in the particular assay. For this, a serial dilution of NBI-74330 was used. Seven different concentrations of NBI-74330 ranging from 10 pM to 10 μM were pipetted in duplicate or triplicate on each 96-well plate after nanofractionation of an analysis mixture and prior to freeze-drying. The last wells in the control rows contained eluent A prior to freeze-drying. After the plates were bioassayed and the bioactivity chromatograms were plotted, a corresponding IC_50_ curve of NBI-74330 was plotted for each 96-well plate from the serial dilution results as a validation of a successful bioassay plate analysis. Subsequently, for each trace, the Z′ factor was calculated as follows:

$$ {Z}^{\prime }=1 - \frac{3{\sigma}_{\mathrm{c}+} + 3{\sigma}_{\mathrm{c}-}}{\left|{\mu}_{\mathrm{c}-}-{\mu}_{\mathrm{c}+}\right|} $$

where the *σ*_c_ refers to the standard deviation of the controls and *μ* refers to the mean of the controls. A value Z′ <0.5 is considered to be an assay with low quality, whereas 1 > Z′ ≥ 0.5 is considered to be an excellent assay, and Z′ = 1 the ideal assay [35]. In our setup, the positive controls (c+) were the wells containing 10 μM NBI-74330, which provided full displacement of the radioligand used. The negative controls (c−) were the wells that contained eluent A prior to freeze-drying. For the outline of the 96-well plates used in this study, please refer to Fig. S1 in the Electronic Supplementary Material (ESM).

The radioligand binding assay was performed in a similar fashion as described previously by Scholten et al. [31]. Shortly, the [^3^H]-VUF11211 radioligand was dissolved in binding buffer (50 mM Tris-HCl buffer pH 7.4 at room temperature, 100 mM NaCl, 0.1 % Tween 80, and 0.1 % BSA) and added to wells containing ligand or collected high-resolution fractions of the ligands. These fractions contained the incubated ligands and/or their metabolites freeze-dried directly on the plate. Subsequently, a suspension of HEK293/CXCR3 membrane extract (7.9 pmol CXCR3 receptor/mg protein) in binding buffer was added to each well, such that the final concentration of protein was approximately 8 μg/well, which corresponded to 63 fmol of CXCR3 receptor [31]. The final concentration of the radioligand in the wells was approximately 2 nM, and the total assay volume was 100 μl/well. After 2 h incubation at room temperature, the membranes were collected on Whatman GF/C filter plates pretreated with 0.5 % (*w*/*v*) BSA in H_2_O and rapidly washed three times with ice-cold wash buffer (50 mM Tris-HCl buffer pH 7.4 at 4 °C, 500 mM NaCl) to remove unbound radioligand. Next, the plates were dried for 30 min at 55 °C followed by addition of 25 μL/well scintillation liquid with a multidrop (Thermo Fisher, Breda, The Netherlands). A PerkinElmer 1450 MicroBeta scintillation counter was used to record the radioactivity on the GF/C plates.

The ^125^I-CXCL10 displacement assay was done in the same fashion. The radioligand was diluted in binding buffer (50 mM HEPES buffer pH 7.4 at room temperature, 500 mM NaCl, 5 mM MgCl_2_, and 1 mM CaCl_2_) to the final concentration of approximately 100 pM. The final concentration of the membrane suspension was 15 μg/well. The GF/C plates were pretreated with 0.5 % (*w*/*v*) PEI in H_2_O and rapidly washed three times with ice-cold wash buffer (50 mM Tris-HCl buffer pH 7.4, 500 mM NaCl, 5 mM MgCl_2_, and 1 mM CaCl_2_).

## Results

### Nanofractionation and assay optimization

An analytical method was developed for structure identification and bioaffinity profiling of metabolic mixtures for the CXCR3 lead compounds NBI-74330 and VUF11211 using the approach depicted in Fig. 1b. A standard mixture of these two small-molecule CXCR3 ligands (20 μM) was used for method optimization and validation. Subsequently, this optimized method was applied to the analysis of metabolic mixtures of these two compounds. These two compounds belonging to different chemical classes were chosen for the optimization of the method considering that the approach developed was optimized with respect to the radioligand binding assay. In general, the LC gradient has to be adjusted to each analysis mixture to obtain satisfying separation of the compounds present in the mixture.

#### Radioligand concentration and membrane amounts

**Fig. 2:**
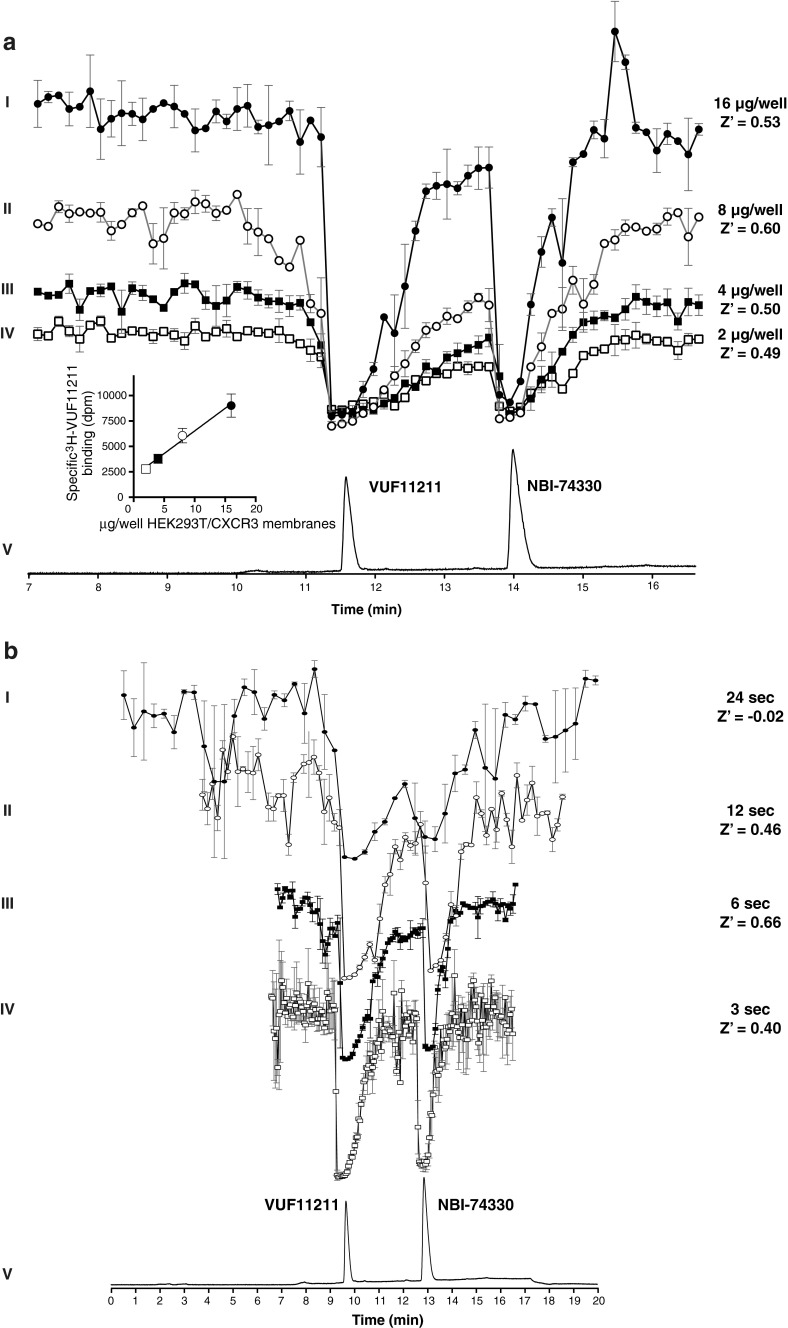
Nanofractionation and assay optimization. **a** The optimization of the concentration of the membrane preparation. Fractions were collected in 12 s/well resolution after 100 μL injection and separation of mixture of NBI-74330 and VUF11211 with 20 μM end concentration per compound. Traces I–IV are reconstructed bioaffinity chromatograms from the radioligand binding assay for CXCR3 receptor binding. UV chromatogram trace (*V*) obtained after injection of 200 μM is given for the correlation with the identity of the compounds. Bioaffinity traces correspond to bioaffinity chromatograms obtained when final concentrations of proteins in the membrane preparation were: 16 μg/well (*I*), 8 μg/well (*II*), 4 μg/well (*III*), and 2 μg/well (*IV*), respectively. The end concentration of ^3^H-VUF11211 radioligand was 2 nM. All bioaffinity chromatograms are scaled equally and shown as an average of a duplicate measurement, where the error bars reflect the variation between the two separate measurements. **b** The optimization of the nanofractionation resolution. Fractions were collected after 100 μL injection and separation of mixture of NBI-74330 and VUF11211 with 20 μM end concentration per compound. Traces I–IV are bioaffinity chromatograms from the radioligand binding assay for CXCR3 receptor binding when four different nanofractionation resolutions were applied. UV chromatogram trace (*V*) obtained after injection of 200 μM is given for the correlation with the identity of the compounds. Traces I–IV correspond to bioaffinity chromatograms obtained with 24, 12, 6, and 3 s/well nanofractionation resolution, respectively. The end concentration of the membrane preparation was 8 μg/well and [^3^H]-VUF11211 radioligand concentration was 2 nM. All bioaffinity chromatograms are scaled equally and shown as an average of a duplicate measurement, where the error bars reflect the variation between the two separate measurements

The method was first optimized for the concentration of [^3^H]-VUF11211 radioligand, the amount of used membranes expressing the CXCR3 receptor, and the nanofractionation resolution after LC separation. The optimal radioligand concentration was selected to be 2 nM per well based on the initial results of the K_d_ determination that was performed for the radioligand characterization [31]. Subsequently, the method was optimized for the amount of membranes expressing CXCR3 that was used per well of the bioassay microtiter plate. A concentration series of membrane preparations (16, 8, 4, and 2 μg/well) was tested in duplicate, and the results are shown in Fig. 2a (12 s/well nanofractionation resolution). A bioaffinity peak was defined as a decrease in signal more than three times the standard deviation of the average baseline and should consist of more than two data points. The peaks from the bioaffinity traces corresponded to the LC-UV trace (Fig. 2a trace V) and were matched to either VUF11211 or NBI-74330. The identity of compounds was confirmed by using accurate mass measurements obtained with high-resolution MS. The two peaks observed for every superimposed chromatogram in Fig. 2a correspond to the two compounds in the standard mixture and are easily detected in all four bioaffinity traces. For all conditions, the Z′ factor was calculated, which is a statistical measure of effect size taking intra-experimental variability into account. The membrane concentration of 8 μg/well was selected for further experiments as it displayed the highest Z′ factor 0.60, suggesting that it is an excellent assay in terms of baseline separation and intra-assay variability (Fig. 2a).

#### Resolution of nanofractionation

The next step in the method development was the optimization of fractionation resolution, which determines the quality of correlation between the chromatographic separation and bioassay readout. Higher nanofractionation frequencies yield more assay points per separated peak, thereby providing better resolution capabilities. This comes with the compromise of sensitivity as separated compounds are fractionated over more wells and are thus more diluted in the bioassay. Nanofractions of the standard mixture were collected in two relatively low (24 and 12 s/well) and two high (6 and 3 s/well) resolution. The resulting bioaffinity chromatograms are shown in duplicate in Fig. 2b. Detection of clear bioaffinity peaks with their accurate correlation to chromatography peaks was not reliably done with 24 s/well fractionations (trace I) due to the low number of assay points describing each peak in combination with relatively high baseline fluctuations (large standard deviation). On the other hand, 3 s/well fractions (trace IV) result in a large number of assay points describing the bioaffinity chromatogram, but at the cost of more diluted fractions, and the inability of collecting a full chromatogram on one 96-well plate, thereby introducing potential interplate differences. The 6 s/well (trace III) and 12 s/well (trace II) fractions produced good resolution and the ability to be performed on one 96-well plate. The 6 s/well fractions resulted in the most optimal assay conditions in terms of the Z' factor (0.66). The overall optimal bioassay conditions were found to be: 2 nM radioligand concentration, 8 μg/well protein concentration in crude membrane suspension, and a nanofractionation frequency of 6 s/well.

#### Evaluation and validation

**Fig. 3:**
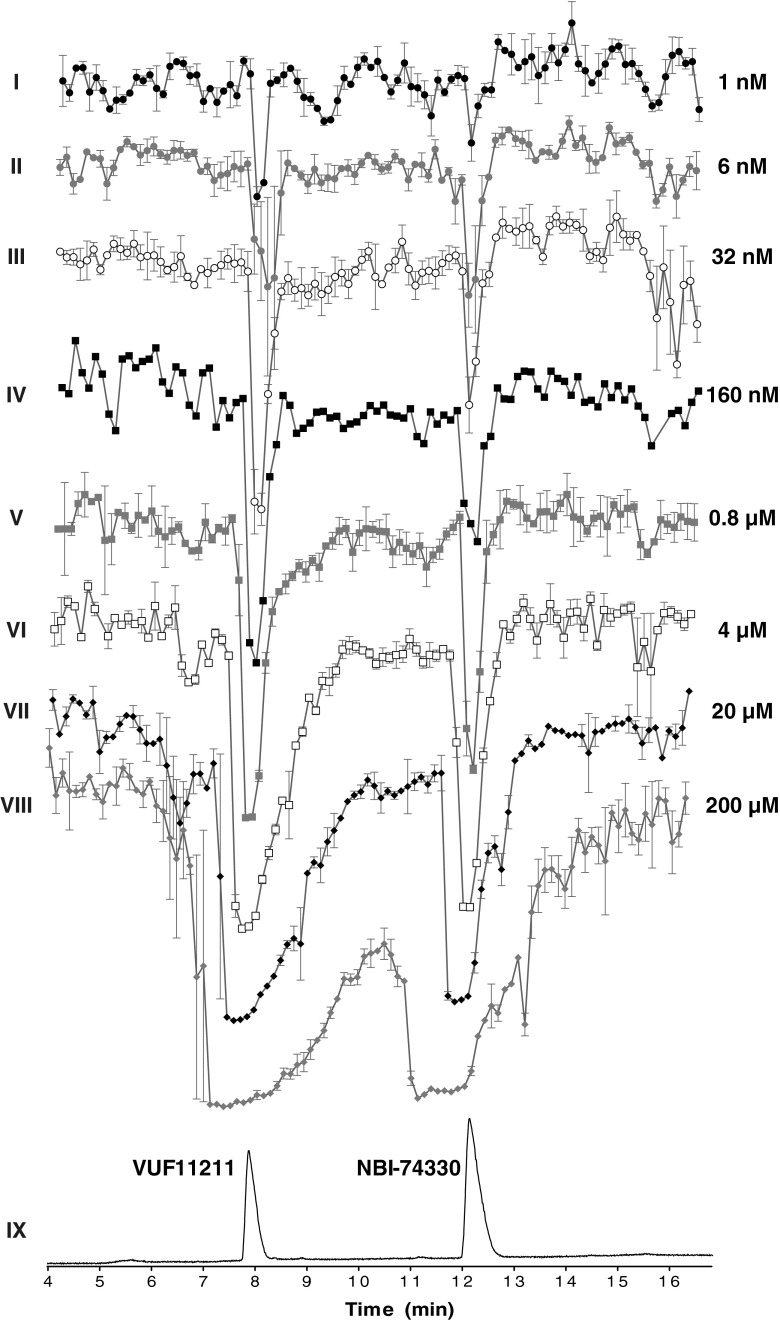
Calibration of the radioligand binding assay. Traces I–VIII correspond to bioaffinity chromatograms obtained after injecting the series of dilutions of the mixture of the two lead compounds (NBI-74330 and VUF11211). The injected concentrations were 1.28, 6.24, 32, 160, and 800 nM and 4, 20, and 200 μM, respectively. All bioaffinity chromatograms are shown as an average of a duplicate measurement. Nanofractionation resolution was 6 s. The end concentration of the CXCR3 membrane preparation was 8 μg/well and [^3^H]-VUF11211 radioligand concentration was 2 nM. UV chromatogram trace (*IX*) obtained after injection of 200 μM is given in *black* for the correlation with the identity of the compounds. All bioaffinity chromatograms are scaled equally and shown as an average of a duplicate measurement, where the error bars reflect the variation between the two separate measurements

Next, in order to evaluate and validate the method with respect to detection sensitivity, the bioaffinity assay was calibrated by using a range of different concentrations of the two compounds in the mixture (1 nM to 200 μM). The results obtained are shown in Fig. 3. The peaks have narrow shapes upon analysis of lower concentrations (traces I–V), while they become broader with flattened tops when higher concentrations of compounds are analyzed (traces VI–VIII). This is inherent to the nature of the bioaffinity response: ligand binding displays a saturation kinetics, which, when represented in log M scale, gives a sigmoidal concentration response curve. This implies that at a certain injected concentration, a full displacement of radioligand is attained, resulting in a peak maximum. Increasing the compound concentration beyond this point only leads to peak broadening as maximum displacement will also be attained in surrounding wells. The bioaffinity traces with increasing peak maxima correspond to the dose-dependent increases in binding of the compounds to the receptor. Concentrations outside this range give either no signal (low concentrations) or maximal signal (high concentrations). In other words, radioligand binding assay represents a bioaffinity detector capable to distinct different concentrations within its linear dynamic range. This is comparable to other detectors, such as MS and UV detectors used in this study. Both MS and UV detectors give sharp peaks within their linear dynamic range, while they cannot make a clear distinction between concentrations that fall outside of this range. The differences in peak narrowness observed for the same concentration of compounds analyzed with different detectors are result of a different sensitivity of these detectors. Thus, the observed peak broadening effects in the bioaffinity trace, which are to some extent also present in the MS, but not observed in the UV chromatogram, come from different sensitivities of the detectors used. This becomes more evident when the limits of detection (LODs) for the two compounds measured with the different detectors used in this study are compared. The LODs fall within low micromolar range for the UV detection (approximately 4 μM for both compounds tested) and in the low nanomolar range for MS (approximately 15 nM injection for both compounds tested) and bioaffinity (approximately 6 nM for NBI-74330 and 1 nM for VUF11211) detection.

**Fig. 4:**
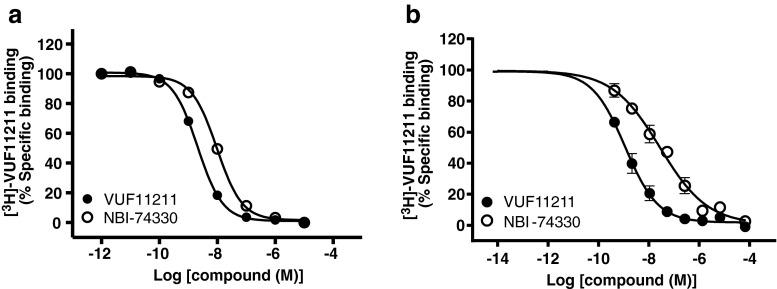
**a** Concentration response curves of NBI-74330 and VUF11211 measured in conventional radioligand binding assay. Binding experiments were performed with approximately 1nM [^3^H]-VUF11211 and increasing concentrations of NBI-74330 (*open circles*) and VUF11211 (*filled circles*). Data are presented as percentage of specific binding. Obtained pK_i_ values were 8.4 ± 0.0 for NBI-74330 and 9.1 ± 0.0 for VUF11211. **b** Concentration response curves of NBI-74330 and VUF11211 constructed from the data obtained during the calibration of the developed method. Each point represents the percentage of [^3^H]-VUF11211 specific binding for each injected concentration of NBI-74330 (*open circles*) and VUF11211 (*filled circles*). The specific binding is defined as the corresponding peak height in the bioaffinity chromatogram. Obtained pK_i_ values were 7.9 ± 0.1 for NBI-74330 and 9.3 ± 0.1 for VUF11211

Results presented in Fig. 3 clearly show the suitability of the developed approach for bioaffinity screening of the small molecule ligands after LC separation and nanofractionation. The LOD for both compounds are in the nanomolar range, as can be seen in Fig. 3, which is in agreement with the affinities of these compounds determined in the conventional [^3^H]-VUF11211 binding assay (pKd = 9.1 ± 0.0 for VUF11211 and pKi = 8.4 ± 0.0 for NBI-74330) as depicted in Fig. 4a (data reproduced from Scholten et al. (32)). Similar nanomolar affinities were also measured in a conventional radioligand binding assay where the ligands were subjected to freeze-drying and reconstitution in assay buffer (data not shown). Using the peak height from the bioassay (Fig. 3), concentration response curves were constructed (Fig. 4b), yielding similar pKi values (pKd = 9.3 ± 0.1 for VUF11211 and pKi = 7.9 ± 0.1 for NBI-74330).

**Fig. 5:**
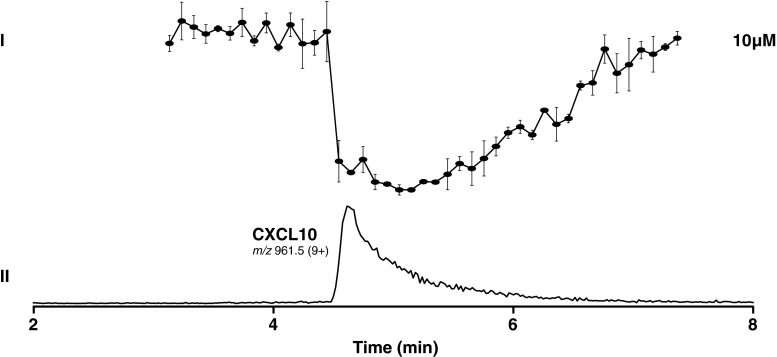
Analysis of a CXCR3 endogenous ligand, CXCL10 chemokine. *I* Bioaffinity chromatograms after LC separation and nanofractionation after duplicate injections of 10 μM CXCL10 solution in water. Fractions were collected in 6 s resolution. The end concentration of the membrane preparation was 10 μg/well and ^125^I-CXCL10 radioligand concentration was 125 pM. The bioaffinity chromatogram is shown as an average of a duplicate measurement. *II* LC-MS trace depicted as extracted ion current (XIC) of CXCL10 after 10 μM solution injection. Correlation between structure identity and bioaffinity is enabled by the parallel bioaffinity and MS analysis

In the abovementioned bioassay setup, a small molecule allosteric radioligand [^3^H]-VUF11211 was used, which binds to the transmembrane domains of CXCR3 [28]. The CXCR3 chemokine CXCL10, the orthosteric ligand, did not produce any displacement of [^3^H]-VUF11211 (not shown) as expected based on results found earlier [31]. To check whether chemokine proteins are compatible with the assay procedure (freeze-drying and fractionation), a control experiment was carried out using ^125^I-CXCL10 as radioligand and CXCL10 as competitor. Using this approach, CXCL10 was injected and nanofractionated for assessment of receptor binding after HPLC separation. The nanofractionated CXCL10 was still able to displace the orthosteric chemokine ^125^I-CXCL10 (Fig. 5), suggesting this approach could potentially be extended to studying proteins.

### Metabolic profiling

**Fig. 6:**
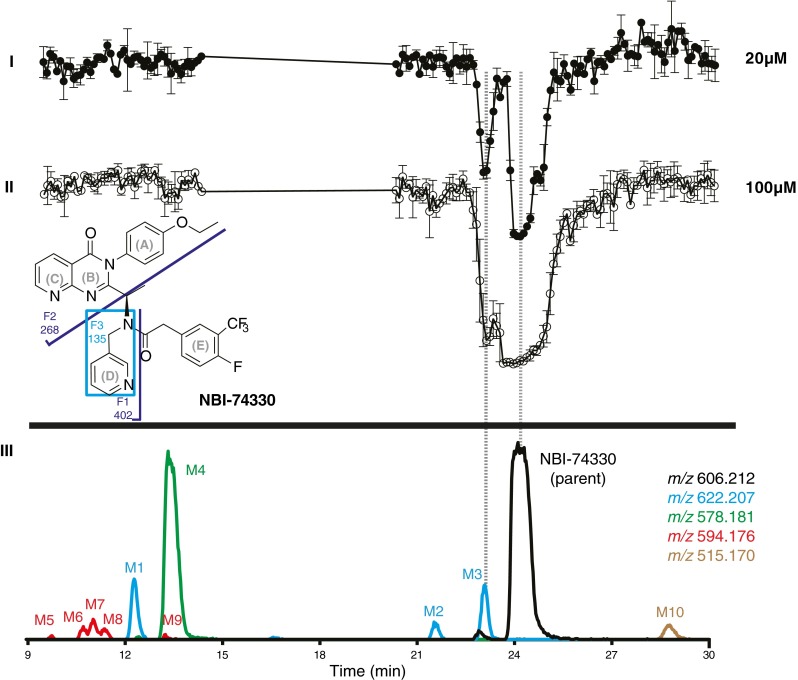
Analysis of a metabolic mixture of NBI-74330, a small molecule ligand towards the CXCR3 receptor. Chemical structure of the parent compound with the MS^2^ fragmentation scheme is inserted in the figure. *I* and *II* Reconstructed bioaffinity chromatograms after LC separation and nanofractionation of the metabolic mixture after duplicate injections at two different concentrations corresponding to 20 and 100 μM pre-incubation concentration of the parent compound. *III* LC-MS traces depicted as extracted ion currents (XICs) of parent compound (*black trace*) and formed metabolites (*color coded notation*). Correlation between structure identity and bioaffinity is enabled by the parallel bioaffinity and MS analysis. All bioaffinity chromatograms are scaled equally and shown as an average of a duplicate measurement, where the error bars reflect the variation between the two separate measurements

**Fig. 7:**
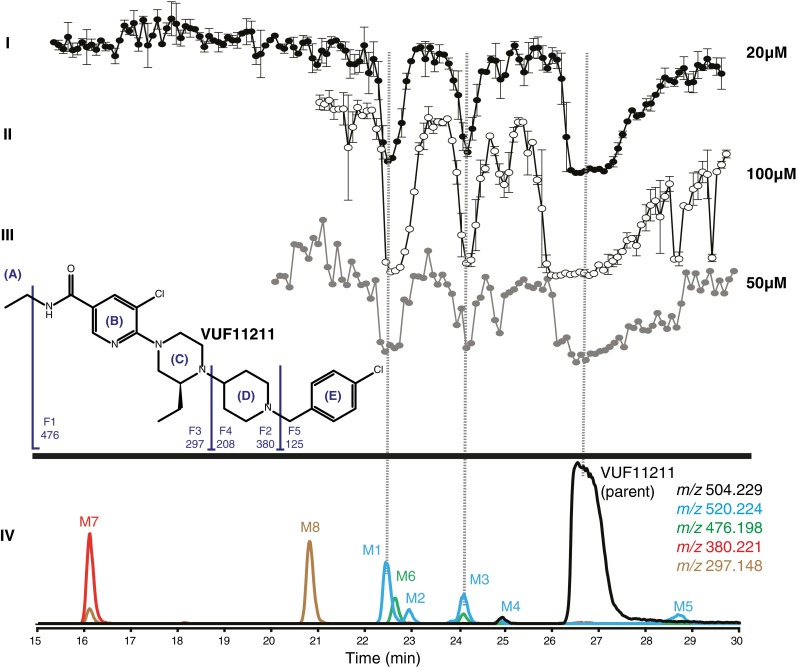
Analysis of a metabolic mixture of VUF11211, a small molecule ligand towards the CXCR3 receptor. Chemical structure of the parent compound with the MS^2^ fragmentation scheme is inserted in the figure. *I* and *II* Reconstructed bioaffinity chromatograms after LC separation and nanofractionation of the metabolic mixture after duplicate injections at two different concentrations corresponding to 20 and 100 μM pre-incubation concentration of the parent compound. *III* Reconstructed bioaffinity chromatogram after LC separation and nanofractionation of the metabolic mixture after duplicate injections at the concentration corresponding to 50 μM pre-incubation concentration of the parent compound. Radioligand binding assay was performed with 125 pM ^125^I-CXCL10 radioligand. *IV* LC-MS traces depicted as extracted ion currents (XICs) of parent compound (*black trace*) and formed metabolites (*color coded notation*). Correlation between structure identity and bioaffinity is enabled by the parallel bioaffinity and MS analysis. All bioaffinity chromatograms are scaled equally and shown as an average of a duplicate measurement, where the error bars reflect the variation between the two separate measurements

The validation of the analytical method was proceeded with the metabolic profiling of the two lead compounds NBI-74330 and VUF11211. After in vitro preparation of the metabolic mixtures, the workup procedure involved reconstitution of metabolic mixtures in eluent A after protein precipitation and evaporation. All the measurements have been done in duplicate using optimized method. The metabolic profiles of NBI-74330 and VUF11211 are shown in Figs. 6 and 7, respectively. The bottom part of both figures represents extracted ion chromatograms (XICs) of parent compound and formed metabolites (metabolite number annotation, with corresponding *m*/*z* values presented in Tables 1 and 2). The upper part of the figures shows reconstructed bioaffinity chromatograms of two different dilutions of the metabolic mixtures injected. As with the parent compounds, the negative peaks reflect binding to CXCR3 measured in the radioligand binding assay. Correlation between structure identity and bioaffinity is enabled by the parallel bioaffinity and MS analysis. Identification of metabolites is based on shifts in accurate mass of parent and formed metabolites and of their fragments in MS^2^. Chemical structures of NBI-74330 and VUF11211 showing their fragmentation patterns in MS^2^ are inserted in Figs. 6 and 7, respectively. Tabulated information on the structure elucidation of the metabolites formed from NBI-74330 and VUF11211 is given in Tables 1 and 2, respectively.

**Table 1:** Interpretation of the MS and MS^2^ results for NBI-74330 and its metabolites M1-M10. F1-F3 are characteristic fragments seen in MS^2^ spectra as depicted in Fig. 6. A–E represent different parts of the structure as depicted in Fig. 6

*m*/*z*	Parent 606.212	F1 402.193	F2 268.108	F3 135.093	Proposed interpretation
Compound	Δ	Δ	Δ	Δ	
Parent	=	=	=	=	Parent compound, NBI-74330
M1	+16	+16	+16	=	+O in ABC
M2	+16	+16	+16	=	+O in ABC
M3	+16	+16	=	+16	+O in D
M4	−28	−28	−28	=	O-dealkylation (change in A)
M5	−28; +16	−28	−28		O-dealkylation, +O in E
M6	−28; +16	−12	−12		O-dealkylation, +O in ABC
M7	−28; +16	−12	−12	=	O-dealkylation, +O in ABC
M8	−28; +16	−12	−12	=	O-dealkylation, +O in ABC
M9	−28; +16	−12			O-dealkylation, +O in ABC
M10	−91	−91	=		N-dealkylation of amide; loss of D

**Table 2:** Interpretation of the MS and MS^2^ results for VUF11211 and its metabolites M1–M8. F1–F5 are characteristic fragments seen in MS^2^ spectra as depicted in Fig. 7. A–E represent different parts of the structure as depicted in Fig. 7

*m*/*z*	Parent 504.229	F1 476.198	F2 380.221	F3 297.148	F4 208.088	F5 125.016	Proposed interpretation
Compound	Δ	Δ	Δ	Δ	Δ	Δ	
Parent	=				=	=	Parent compound, VUF11211
M1	+16				=	=	+O in ABC
M2	+16			=			+O in E
M3	+16	=					+O in A
M4	+16				+16		+O in DE
M5	+16				=		+O in ABC
M6	−28	=			=		N-dealkylation of amide (- C_2_H_4_ from A)
M7	−124		=	=			N-dealkylation of piperidine, loss of E
M8	−207			=			N-dealkylation of piperazine, loss of DE

#### Metabolic profiling of lead compound NBI-74330

Two peaks were observed in the bioaffinity chromatogram of the metabolic mixture of NBI-74330 (Fig. 6(I and II)). These peaks corresponded to the parent compound and Metabolite 3. In total, ten metabolites were detected in the metabolic mixture of NBI-74330 (Fig. 6(III)). Five primary metabolites were: Metabolites 1, 2, and 3, which had a shift of *m*/*z* +16 indicating oxidation in the molecule, Metabolite 4, which had a shift of *m*/*z* –28 indicating *O*-deethylation of the phenol ring, and Metabolite 10, which had a shift of *m*/*z* –91 pointing to *N*-dealkylation of the amide (removal of methylpyridyl). Secondary metabolism products, Metabolites 5–9, all had a shift of *m*/*z* –12, which indicated that *O*-dealkylation (loss of the ethyl group in the phenol ring) in combination with an oxidation. The structures of Metabolite 3, 4, and 10 could be completely elucidated, while for the other metabolites only partial identification was achieved. The single-charge precursor ion [M + H]^+^ of NBI-74330 with *m*/*z* 606.212 gives three major fragments in MS^2^ mode: ions with *m*/*z* 135.093, 268.108, and 402.193. The proposed structures of these fragments are shown in Fig. 6. The single charged ion of Metabolite 3, *m*/*z* 622.207, gave three major fragment ions in the MS^2^ spectra with *m*/*z* 151.088, 268.108, and 418.187, which indicated oxidation in the pyridyl-methyl moiety. A closer look at the MS data revealed three small ions corresponding to losses of 18, 17, and 16 Da, which are characteristic for *N*-oxides and therefore lead to the conclusion that Metabolite 3 is the pyridyl-*N*-oxide of NBI-74330. Metabolite 3 also showed bioaffinity towards CXCR3 (see Fig. 6). These conclusions are consistent with the findings of Jopling et al. [32] who used a more elaborate approach to analyze the bioaffinity of NBI-74330 and its major metabolites. Furthermore, a bioactive pyridyl-*N*-oxide metabolite is also formed during metabolism of AMG-487, a structural analog of NBI-74330. Metabolite 4, *m*/*z* 578.181, with a −28 Da shift compared to the parent molecule gave three fragments in MS^2^ spectra with *m*/*z* 135.093, 240.077, and 374.161, indicating *O*-deethylation. Metabolite 10, *m*/*z* 515.170, showed a −91 Da shift, corresponding to *N*-dealkylation of the amide and loss of the pyridinyl-methyl group, which is supported by the fragment ions with *m*/*z* 268.108 and 311.151 in its MS^2^ spectrum.

#### Metabolic profiling of lead compound VUF11211

In the bioaffinity chromatogram of the metabolic mixture of VUF11211, three negative peaks were observed which corresponded to the parent compound and Metabolite 1 and Metabolite 3 (Fig. 7(I and II)). In addition to this [^3^H]-VUF11211 based profiling, for proof of principle, the ability of metabolites to displace the natural orthosterically binding chemokine was also demonstrated using a ^125^I-CXCL10-based radioligand binding assay. As shown in Fig. 7(III), next to the parent compound, both metabolites were shown to displace the natural chemokine CXCL10. The developed setup enabled detection of eight metabolites in the metabolic mixture. All eight metabolites detected were primary biotransformation products: Metabolites 1–5, which had a shift of *m*/*z* +16 indicating oxidation in the molecule, Metabolite 6, which had a shift of *m*/*z* −28 indicating *N*-deethylation of the amide, Metabolite 7, which had a shift of *m*/*z* −124 pointing at *N*-dealkylation of the piperidine ring (removal of 4-chloro toluene), Metabolite 8, which had a shift of *m*/*z* −207 that indicated *N*-dealkylation of the piperazine ring (removal of 1-(4-chlorobenzyl) piperidine). Metabolites 1 and 3 were found to have affinity for CXCR3. Structure elucidation of the VUF11211 metabolites was complicated due to the poor fragmentation pattern of the parent compound. VUF11211 gave only two fragments coming from the same part of the molecule and thus restricted structural information on the rest of the molecule. At the same time, small changes in the original structure upon biotransformation yielded completely different fragmentation patterns for some of the metabolites. Full structure identification was obtained for Metabolites 6–8, while the other structures were only partially elucidated. The single-charge precursor ion [M + H]^+^ of the parent with *m*/*z* 504.229 gives two major fragments in MS^2^ mode: *m*/*z* 125.016 and 208.088, both having a characteristic chlorine pattern. The proposed structures of these fragments are shown in Fig. 7, together with the proposed structures of the fragments that were obtained in MS^2^ spectra of some of the metabolites but not of the parent compound itself. Metabolite 1, *m*/*z* 520.223, had a shift of *m*/*z* +16 and gave two major fragments in the MS^2^ spectra corresponding to ions with *m*/*z* 125.016 and 208.089, which indicated oxidation in the ABC ring of the original molecule (as annotated in Fig. 7 concerning the profile groups in the structure of the compound). Metabolite 2, *m*/*z* 520.223, with a shift of *m*/*z* +16 compared to the parent compound, gave a fragment with a mass of *m*/*z* 380.220 pointing to oxidation in the benzyl ring (ring E). The precursor ion of Metabolite 3 with *m*/*z* 520.223 gave one fragment in MS^2^ spectra with *m*/*z* 476.192 (loss of 44.031 Da), which led to the conclusion that the oxidation took part on the ethyl moiety of the amide group. Metabolite 4 with *m*/*z* 520.219 gave one fragment in MS^2^ spectra with *m*/*z* 224.081, which indicated oxidation in the DE ring, while Metabolite 5 with *m*/*z* 520.222 gave a fragment with *m*/*z* 208.087 indicating the oxidation to be in the ABC part. Metabolite 6, *m*/*z* 476.198, had a mass shift of −28 Da compared to the parent compound. Only one fragment ion with *m*/*z* 208.092 was found in the MS^2^ spectra of Metabolite 6. *N*-Deethylation of the amide group is proposed to be the metabolic conversion leading to formation of Metabolite 6. Metabolite 7, *m*/*z* 380.221, and Metabolite 8, *m*/*z* 297.148, did not give significant fragmentation, but the mass shifts of 124 and 207 Da, respectively, clearly indicated *N*-dealkylation of the parent molecule in both cases: Metabolite 7 corresponds to *N*-dealkylation of piperidine ring (removal of 4-chlorophenylmethyl group) and Metabolite 8 to *N*-dealkylation of the piperazine ring (removal of 1-(4-chlorobenzyl)piperidinyl group).

## Discussion

In this paper, a recently developed analytical methodology for the bioaffinity profiling of mixtures of compounds targeting the histamine receptor was transferred, optimized, and applied for bioaffinity assessment of mixture of ligands targeting the CXCR3 receptor. This method encompasses LC separation of a mixture of compounds, MS detection, and subsequent structure elucidation of each compound and a parallel radioligand binding assay for bioaffinity assessment. Furthermore, the comprehensive metabolic profiling of the important CXCR3 lead compounds NBI-74330 and VUF11211 with the optimized method is reported. This method allows for efficient and rapid metabolic profiling of metabolic mixtures of small ligands towards the CXCR3 chemokine receptor, including bioaffinity assessment. Successful combination of analytical techniques and high-resolution at-line nanofractionation with a CXCR3 radioligand binding assay was demonstrated. This analytical platform allows for separation of the different compounds in a metabolic mixture and their direct subsequent bioaffinity analysis. Parallel accurate mass measurements in MS and MS^2^ mode allow (partial) structure elucidation of the formed metabolites with correlation to their bioaffinity. In this way, metabolic profiling of lead compounds and potential drug candidates is enabled in a rapid and comprehensive manner. The method provides a quick snapshot of metabolic profiles of lead compounds for the CXCR3, including their bioaffinity, and thereby facilitates this type of profiling earlier on in drug discovery, to be used for larger sets of interesting compounds in a drug discovery pipeline.

Metabolic profiling represents a very important step in the drug discovery process, as this is a first step in the determination of a pharmacokinetics, bioactivity, and selectivity profile of a compound. However, due to the need for expensive and laborious methods, metabolic profiling is generally applied when a compound is in a more advanced stage of discovery process. Usually, the metabolic stability is assessed first. At a later stage, when a lead compound successfully passes metabolic stability tests, the formed metabolites are identified, and bioactivity and selectivity of metabolites are determined. An early-stage metabolic (bioactivity) profiling of lead compounds and drug candidates could cut the expenses of developing a drug by rapid assessment of undesirable metabolic profiles. New analytical approach allows combining these analyses in one analytical method and, as such, simultaneously yields rapid snapshots on metabolic stability and bioaffinity of the formed metabolites. This was demonstrated on the example of two well-known CXCR3 ligands, NBI-74330 and VUF11211. The new optimized method allowed identification and (partial) structure elucidation of 9 metabolites of NBI-74330 and 8 metabolites of VUF1121. These metabolites, with the exception of the pyridyl *N*-oxide of NBI-74330, have not been described previously. Furthermore, metabolite M3 of NBI-74330 and metabolites M1 and M3 of VUF11211 showed to possess bioaffinity for CXCR3 receptor. However, the method developed gives only relative estimation of the binding affinities of the metabolites formed. On the example of VUF11211 and NBI-74330, it was shown that the method can detect high affinity binders even if they are present in very low concentration, close to their IC_50_ values. Under the conditions used for metabolic incubations in this study, only a small amount of parent compound (generally 1–5 %) is converted into the metabolites; thus, the concentration of the metabolites in the metabolic mixture is very low. Therefore, it is estimated that the metabolites that bind to the CXCR3 receptor represent the high affinity binders, such as metabolite M3 of NBI-74330 and metabolites M1 and M3 of VUF11211. All the other metabolites present could have no affinity for CXCR3 receptor or they could be low affinity binders. The real determination of the bioaffinity of each metabolite demands synthesis or purification of the metabolites followed by determination of their pK*i* values. A rough estimation of the K*i* value could be made if the metabolite concentrations are known. A combination of known responses in UV absorption and MS ionization efficiency from the mother compound with the rationale that metabolites with similar structures often give similar responses can however give an estimate of the abundance of each metabolite in a mixture, but never absolute quantification can be done. This rationale together with the bioaffinity signal is used to depict a metabolite as low-affinity or high-affinity binder.

At the same time, provided that metabolite elucidation is sufficiently possible, studying the bioaffinity of the metabolites can give valuable information on affinity hotspots in the original molecules. In the current paper, this was indicated with NBI-74330 and VUF11211. For example, the findings on NBI-74330 overlap with results found for its analog AMG 487. It entered clinical phase II trials for psoriasis, but was abandoned due to lack of efficacy, which was attributed to high variability in drug exposure. Later, metabolic studies of AMG 487 in healthy volunteers led to the suggestion that these variations might be due to inhibition of metabolism caused by formation of *O*-deethylated AMG 487 and its sequential secondary metabolites [21, 22]. The *O*-deethylated form of AMG487 (M4) was also found for the closely related compound NBI-74330 (Fig. 6). In the case of AMG487, this metabolite was shown to have a lesser ability to displace the radioligand compared to the parent compound, which provides a likely explanation for the observation that M4 does not displace [^3^H]-VUF11211 in the assay used in this study. Moreover, another known AMG487 metabolite, a pyridyl *N*-oxide, is four times more potent than the parent compound [36], which is qualitatively in line with the observed displacement of analogous metabolite M3 in Fig. 6. This indeed suggests that M3 binds to the CXCR3 receptor. In this study, different known metabolites have been found as well, validating the use of the current method for the screening and characterization of metabolites originating from small-molecule chemokine receptor ligands.

The approach was also applied to VUF11211, for which, to date, no metabolites were described in literature. The discovered metabolites provide insight in which parts of the molecule play a role in affinity of the compound for the CXCR3 receptor. For example, the findings of this study underscore the known importance of the benzyl-piperazine moiety (ED-part in Fig. 7), as its removal (M8) led to undetectable displacement of [^3^H]-VUF11211. On the other hand, the metabolite M3 showed maximum radioligand displacement (Fig. 7), suggesting that oxidized ethyl moieties as amide substituents may pose a viable strategy. Indeed, such side chains have been published by the inventors of VUF11211 [26]. As such, the developed method complements the data obtained through site-directed mutagenesis and receptor modeling studies and serves as additional input for medicinal chemistry programs. Moreover, the presented method would allow for the coupling of HPLC separation and nanofractionation to, e.g., cytochrome P450 inhibition assays to assess toxicological profiles. In addition, it was also demonstrated that an analytical approach as presented in this study can also be used for characterization of chemokine proteins or even derived fragments of chemokines. Modified chemokines for example appear to act as antagonists for certain chemokine receptors and may be valuable as biopharmaceuticals.

Altogether, the analytical techniques described in this study efficiently combine metabolic profiling and chemokine receptor ligand binding assays using nanofractionation as a linking technology. Moreover, the developed method allows the identification of liable hotspots in lead compounds. Hence, such combined approach allows implementation of comprehensive metabolic profiling and directs lead optimization of drug candidates in an early phase of the drug discovery process.

## Electronic supplementary material

ESM 1 (PDF 48 kb)
