# Supplementary material for: Metabolic profiling of ligands for the chemokine receptor CXCR3 by liquid chromatography-mass spectrometry coupled to bioaffinity assessment
Source: Anal Bioanal Chem. 2015 Jul 12;407(23):7067–81. doi: 10.1007/s00216-015-8867-z (PMC4551560; doi:10.1007/s00216-015-8867-z)

Analytical and Bioanalytical Chemistry

Electronic Supplementary Material

Metabolic profiling of ligands for the chemokine receptor CXCR3 by liquid chromatography-mass spectrometry coupled to bioaffinity assessment

Marija Mladic, Danny J. Scholten, Maikel Wijtmans, David Falck, Rob Leurs, Wilfried M.A. Niessen, Martine J. Smit, Jeroen Kool

Fig. S1 Outline of the experiment performed on each 96-well plate. (A) Nanofractions are collected onto a 96-well plate in serpentine fashion. (B) A serial dilution of a control compound ranging from 10 pM to 10 µM is pipetted onto the 96-well plate in triplicate. The plate is subsequently freeze-dried and the radioligand binding assay is performed. The results of the radioligand binding assay are then plotted as a reconstructed bioaffinity chromatogram of a mixture analyzed (C) or a concentration-response curve of the control compound (D). The radioligand binding results from the maximum radioligand displacement (c+) and from no radioligand displacement (c-) are used to calculate the z' factor for each experiment

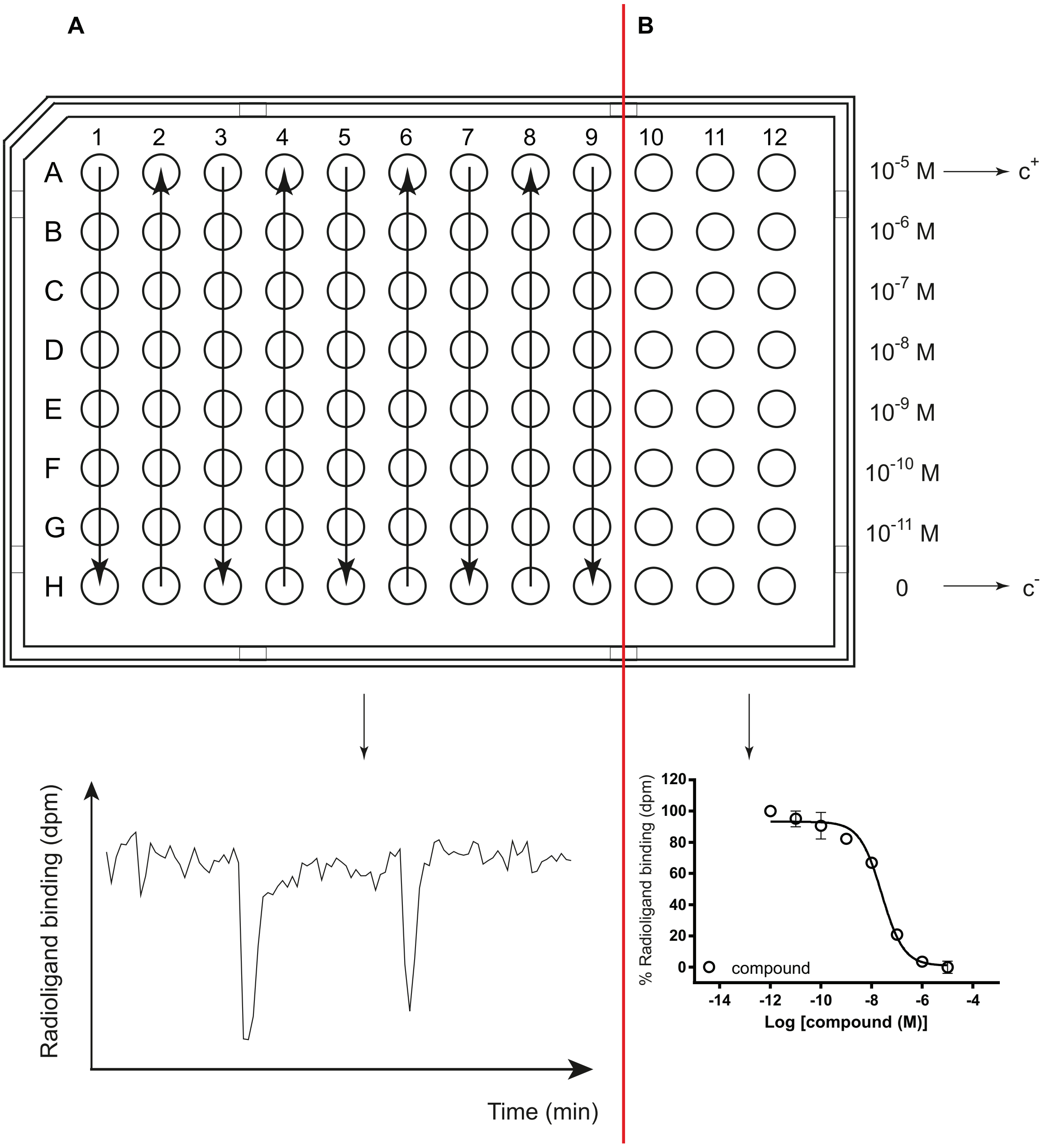

Supplement: Supplementary file 1 — (PDF 48 kb) [file 216_2015_8867_MOESM1_ESM.pdf]
